# Manipulating the Expression of Glycogen Phosphorylase in *Synechococcus elongatus* PCC 7942 to Mobilize Glycogen Storage for Sucrose Synthesis

**DOI:** 10.3389/fbioe.2022.925311

**Published:** 2022-07-01

**Authors:** Yu Dan, Jiahui Sun, Shanshan Zhang, Yannan Wu, Shaoming Mao, Guodong Luan, Xuefeng Lu

**Affiliations:** ^1^ College of Life Science and Technology, Central South University of Forestry and Technology, Changsha, China; ^2^ Hunan Provincial Key Laboratory of Forestry Biotechnology, Central South University of Forestry and Technology, Changsha, China; ^3^ Qingdao Institute of Bioenergy and Bioprocess Technology, Chinese Academy of Sciences, Qingdao, China; ^4^ Shandong Energy Institute, Qingdao, China; ^5^ Qingdao New Energy Shandong Laboratory, Qingdao, China; ^6^ College of Life Science, University of Chinese Academy of Sciences, Beijing, China; ^7^ Laboratory for Marine Biology and Biotechnology, Qingdao National Laboratory for Marine Science and Technology, Qingdao, China

**Keywords:** cyanobacteria, glycogen, sucrose, glycogen phosphorylase, *Synechococcus elongatus* PCC7942

## Abstract

Cyanobacteria are a promising photosynthetic chassis to produce biofuels, biochemicals, and pharmaceuticals at the expense of CO_2_ and light energy. Glycogen accumulation represents a universal carbon sink mechanism among cyanobacteria, storing excess carbon and energy from photosynthesis and may compete with product synthesis. Therefore, the glycogen synthesis pathway is often targeted to increase cyanobacterial production of desired carbon-based products. However, these manipulations caused severe physiological and metabolic impairments and often failed to optimize the overall performance of photosynthetic production. Here, in this work, we explored to mobilize the glycogen storage by strengthening glycogen degradation activities. In *Synechococcus elongatus* PCC 7942, we manipulated the abundances of glycogen phosphorylase (GlgP) with a theophylline dose-responsive riboswitch approach, which holds control over the cyanobacterial glycogen degradation process and successfully regulated the glycogen contents in the recombinant strain. Taking sucrose synthesis as a model, we explored the effects of enhanced glycogen degradation on sucrose production and glycogen storage. It is confirmed that under non-hypersaline conditions, the overexpressed *glgP* facilitated the effective mobilization of glycogen storage and resulted in increased secretory sucrose production. The findings in this work provided fresh insights into the area of cyanobacteria glycogen metabolism engineering and would inspire the development of novel metabolic engineering approaches for efficient photosynthetic biosynthesis.

## Introduction

Cyanobacteria are important prokaryotic microorganisms performing oxygenic photosynthesis and playing essential roles in global carbon and nitrogen cycles on Earth (Waterbury et al., 1979; Flombaum et al., 2013; Rousseaux and Gregg, 2014). Being abundant and widely distributed among diverse types of habitats, cyanobacteria provide 20–30% of the primary organic carbon in the biosphere, with their efficient oxygenic photosynthesis systems converting solar energy and carbon dioxide into organic compounds (Flombaum et al., 2013; Rousseaux and Gregg, 2014). In cyanobacteria cells, the surplus carbon flow beyond the requirements of cellular growth and maintenance would be stored as a carbon sink, supporting materials and energy for the cells to survive in dark and stressful conditions (Ball and Morell, 2003; Nakamura et al., 2005; Damrow et al., 2016). Glycogen is the most essential carbon sink compound in cyanobacteria, accounting for up to 50% of the total cellular biomass in specific species or environments (Aikawa et al., 2014; Song et al., 2016).

**FIGURE 1:**
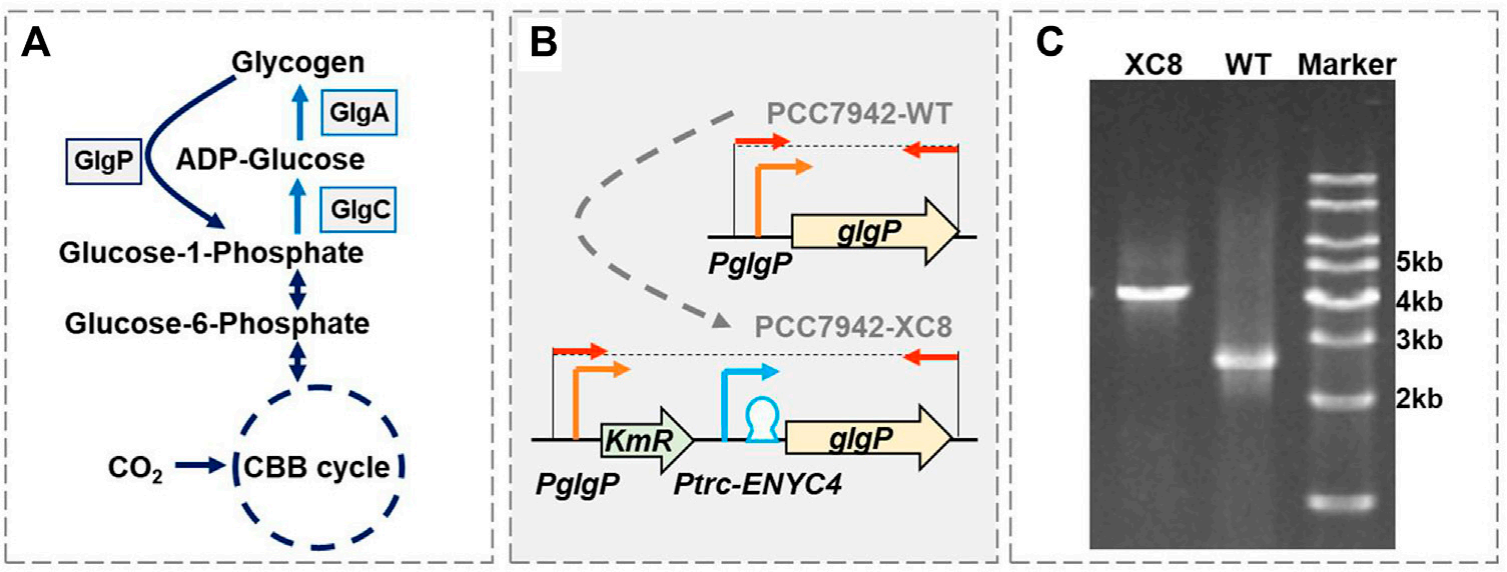
Construction of a theophylline-responsive *glgP* expression system in *Synechococcus elongatus* PCC 7942. **(A)** Glycogen metabolism pathways in cyanobacteria. GlgP, glycogen phosphorylase; GlgA, glycogen synthase; GlgC, glucose-1-phosphate adenylyltransferase or ADP-glucose pyrophosphorylase; CBB cycle, Calvin–Benson–Bassham cycle. **(B)** Strategy for constructing the theophylline-responsive riboswitch control system for *glgP* expression in PCC 7942. A kanamycin resistance gene (KmR) and theophylline dose-regulated expression cassette (*Ptrc-ENYC4*) were inserted between the native promoter of *glgP* (*PglgP*) and the *glgP* CDS, and the obtained mutant was termed as PCC 7942 XC8. The red arrows represented the primer pairs used for checking the genotypes of PCC 7942 wildtype (PCC7942-WT) and XC8 mutant (PCC7942-XC8) strains. **(C)** Identification of the genotypes of PCC 7942 wildtype (WT) and XC8 mutant by PCR with the primers (shown as red arrows in B).

As promising photosynthetic microbial platforms for biotechnological and industrial applications (Angermayr et al., 2009; Lu, 2010; Oliver and Atsumi, 2014; Zhou et al., 2016; Luan and Lu, 2018), diverse strategies and tools have been developed to manipulate carbon flow in cyanobacteria, and glycogen metabolism has been generally recognized as a promising target (Carrieri et al., 2012; Melis, 2012; Zhou et al., 2016; Luan et al., 2019). Metabolic pathways for glycogen synthesis and degradation have been clearly deciphered in cyanobacteria (Figure 1A) and inhibition of the key enzymes for glycogen synthesis, and GlgC (ADP-glucose pyrophosphorylase, catalyzing ADP-glucose formation with glucose-1-phosphate) and GlgA (glycogen synthase, incorporating glucose monomers into the growing 1-4 α-linked glucose polymer) have successfully facilitated effective regulation of glycogen storages (Carrieri et al., 2012; Melis, 2012; Zhou et al., 2016) and intracellular carbon distribution (Xu et al., 2013; Hendry et al., 2017). However, impaired glycogen synthesis usually causes severe disturbance to cell physiology, including reduced photosynthesis, growth, respiration, and robustness toward environmental stresses (Miao et al., 2003; Suzuki et al., 2010; Grundel et al., 2012; Guerra et al., 2013; Hickman et al., 2013). In many cases, blocking glycogen synthesis decreased rather than increasing the productivity of heterologous pathways in engineered cyanobacterial strains, which might be resulted from the combined effect of physiological damage and metabolic rebalance (Davies et al., 2014; Jacobsen and Frigaard, 2014; Li et al., 2014; van der Woude et al., 2014; Work et al., 2015).

Since elimination of the glycogen synthesis flux failed to optimize the photosynthetic biosynthesis performances of the cyanobacterial cell factories, strengthening the glycogen degradation pathways to mobilize the carbon sink into central metabolism could undoubtedly be an alternative strategy worth trying (Luan et al., 2019). However, approaches and effects to manipulate glycogen degradation metabolism in cyanobacteria have been relatively less reported. As shown in Figure 1A, glycogen phosphorylase (encoded by *glgP*), catalyzing the glycogen digestion into glucose-1-phosphate (G-1-P), is mainly responsible for glycogen degradation in cyanobacteria (Fu and Xu, 2006). In 2012, Ducat et al. overexpressed *glgP* in an engineered strain of *Synechococcus elongatus* PCC 7942 for secretory sucrose production and reported that the additional cassette for *glgP* expression caused a 10% reduction in sucrose productivity (Ducat et al., 2012). In general, the possibilities to engineer and accelerate glycogen degradation and the subsequent effects on cellular physiology and metabolism in cyanobacteria are yet to be explored.

In the present study, we aimed to manipulate glycogen metabolism by regulating the expression of *glgP* and the activities of glycogen degradation. Adopting a theophylline dose-responsive riboswitch, GlgP abundances, glycogen phosphorylase activities, and glycogen storage in a model cyanobacterium *Synechococcus elongatus* PCC 7942 (hereafter PCC 7942 for short) were successfully regulated, and the relative effects on cellular physiology and metabolism were explored. Sucrose is another important type of carbohydrate in PCC 7942 to be accumulated as osmoprotectants in response to extracellular hypersaline stress (Hagemann, 2011). In the past years, interactions between sucrose synthesis and glycogen metabolism have been explored in different cyanobacteria species (Suzuki et al., 2010; Guerra et al., 2013; Xu et al., 2013), and several glycogen metabolism engineering strategies have been adopted to improve the production of sucrose and other metabolic products in engineered cyanobacterial cell factories (Ducat et al., 2012; Qiao et al., 2018; Luan et al., 2019). Thus, sucrose synthesis was also taken as a model to explore whether the strategy of accelerating glycogen degradation could enhance the performance of photosynthetic biosynthesis. Our findings provided fresh insights into the area of cyanobacteria glycogen metabolism engineering and would inspire the development of novel metabolic engineering approaches for efficient photosynthetic biosynthesis.

## Results and Discussion

### Construction of a Theophylline-Responsive *glgP* Expression System in PCC 7942

As shown in Figure 1A, glycogen phosphorylase (GlgP) plays an essential role in catalyzing the glycogen degradation process by breaking the α-1,4-glycosidic bond on the glycogen chain and removing glucose monomers in the form of glucose-1-phosphate (G-1-P). It has been confirmed that the knockout of two *glgP* genes in *Synechocystis* sp. PCC 6803 would increase the glycogen contents by about 7% with continuous illumination and 250%–450% in day–night cycles (Shimakawa et al., 2014). However, so far, there have not been reports about quantitatively evaluating the effects of enhanced *glgP* expression on glycogen storage in cyanobacteria. We designed to regulate the expression of *glgP* and glycogen degradation activities adopting a theophylline-dose responsive riboswitch system ENYC4 in PCC 7942. Previously the *Ptrc-ENYC4* system (*Ptrc* promoter combined with the ENYC4 riboswitch sequence) had been successfully utilized to regulate the expression of *glgC* and facilitated dynamic regulation of GlgC abundances and glycogen contents (Qiao et al., 2018; Chi et al., 2019). In this work, we adopted a similar strategy by replacing the native *glgP* promoter sequence with a *Ptrc-ENYC4* sequence (Figures 1B,C). As designed, the expression of *glgP* on the chromosome of PCC 7942 would be regulated by the dose of theophylline.

**FIGURE 2:**
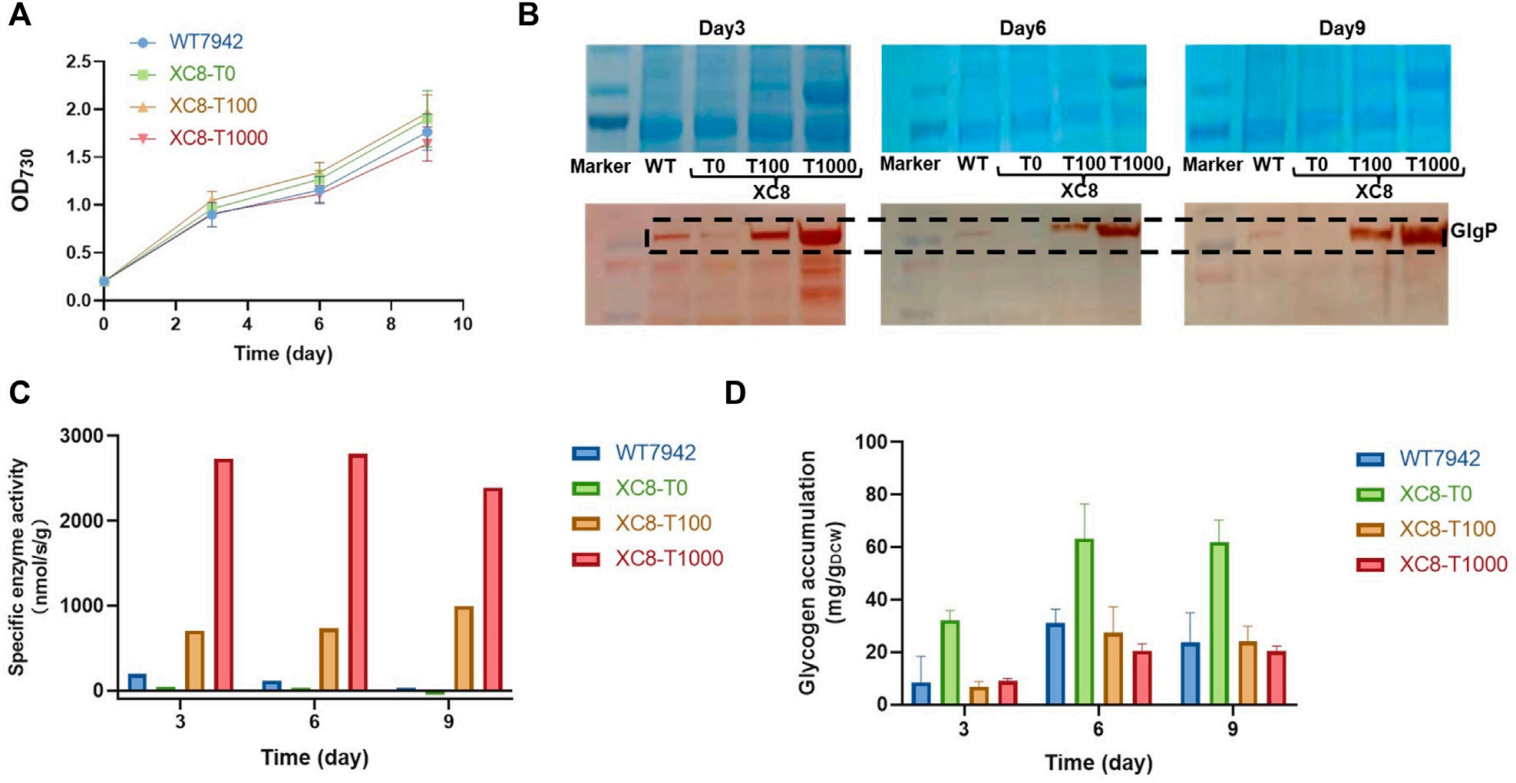
Theophylline-regulated *glgP* expression and glycogen storage in PCC 7942. **(A)** Cell growths, **(B)** GlgP abundances, **(C)** glycogen phosphorylase activities, and **(D)** glycogen contents of XC8 strain induced with different concentrations of theophylline during the 10 days of cultivation. To confirm the effects of theophylline induction on *glgP* expression regulation, different concentrations of theophylline (0 μM, T0; 100 μM, T100; 1,000 μM, T1000) were supplemented into culture broth of XC8 strain in flasks. The cells would be sampled on Day 3, Day 6, and Day 9 for GlgP Western blot assays, glycogen phosphorylase activity calculations, and glycogen content determination.

To evaluate the effects of the theophylline-responsive riboswitch on regulating *glgP* expression, concentrations of theophylline (0,100, and 1,000 μM) were supplemented into the culture broth of XC8, and the abundances of GlgP were evaluated by immunoblotting. As shown in Figure 2A; Supplementary Figure S1, supplementation of theophylline with all of the three concentrations into the culture medium caused minor influence on growths of the XC8 strain, showing similar growth patterns with the wildtype control. During the process, the GlgP abundances were significantly regulated by the addition of theophylline (Figure 2B). Samples were collected from three time points of the cultivation process, and Western blot with GlgP-specific antibodies was performed. When no theophylline was added, the GlgP abundances were significantly reduced to an undetectable level compared with those of the control. The addition of theophylline (100 and 1,000 μM) as an inducer would elevate the expressions of GlgP in XC8. In the medium containing 100 μM theophylline, GlgP abundances would significantly exceed those of the wildtype, and increasing the theophylline concentration to 1,000 μM would further improve the GlgP abundances. The results indicated that in PCC 7942 recombinant strain XC8 carrying the *Ptrc-ENYC4-glgP* cassette, the abundances of GlgP could be effectively regulated with theophylline dose added in the culture broth. In addition, the change in GlgP abundances also brought in consistent changes in glycogen phosphorylase activities in the XC8 strain with increasing theophylline inductions (Figure 2C). With 100 μM theophylline induction, glycogen phosphorylase activities were increased to nearly 4-fold higher than those of the wildtype control.

### Glycogen Storage is Negatively Correlated With the Theophylline-Regulated GlgP Abundances in PCC 7942

It has been confirmed that the GlgP abundances and activities could be artificially regulated through theophylline dose in the XC8 strain; thus, we further determined the influence of artificially regulated glycogen phosphorylase activities on glycogen storage in *Synechococcus* cells. Intracellular glycogen contents of XC8 and wildtype control were calculated on Day 3, Day 6, and Day 9 of the flask cultivation process (Figure 2D). As expected, intracellular glycogen contents of the recombinant strain showed a significantly negative relationship with the theophylline dose and GlgP abundances. Glycogen contents in the wildtype PCC 7942 cells were maintained in the range between 10 mg/gDCW and 30 mg/gDCW. While for the XC8 recombinant strain, when no theophylline was added (meaning that the translation of *glgP* transcripts was still inhibited), the intracellular contents were increased by 2–3 folds compared with those of the wildtype control. When theophylline was supplemented in the culture broth, glycogen storage in XC8 cells would be correspondingly reduced. However, it is also noteworthy that although the GlgP abundances and activities could be increased by theophylline supplementation to levels much higher than those of the wildtype control, the glycogen contents would still be maintained on a normal physiological level similar to the wildtype control.

**FIGURE 3:**
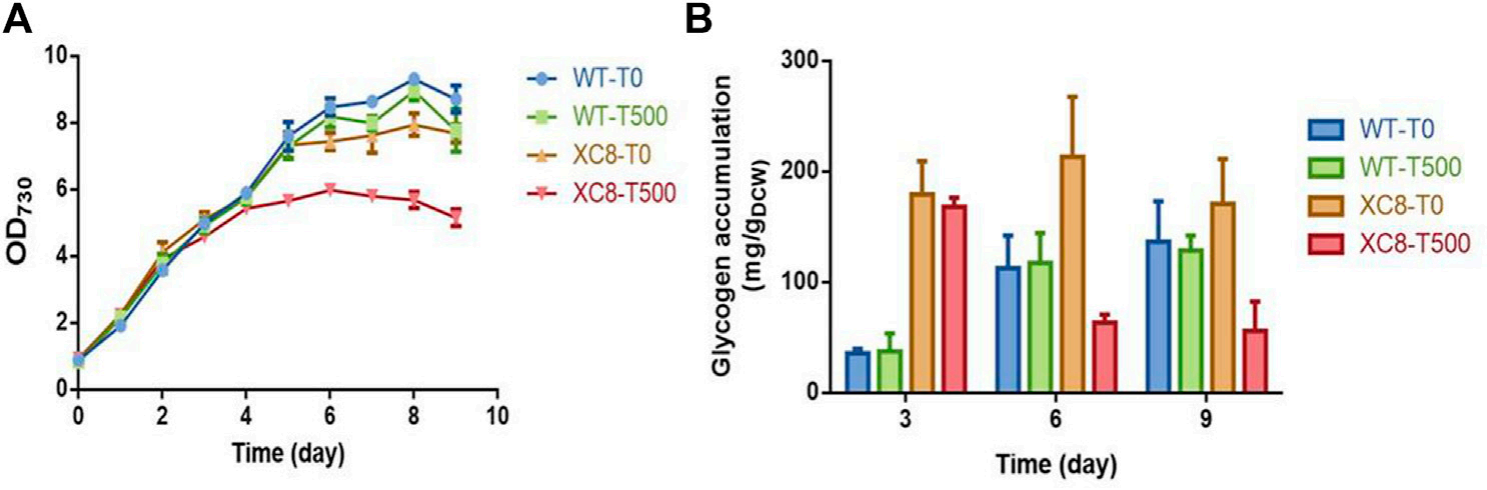
Effects of theophylline-regulated *glgP* expression on PCC 7942 cell growths and glycogen contents in column photobioreactors. **(A)** Cell growth and **(B)** glycogen contents of the wildtype and XC8 strains when cultivated in column bioreactors. The cultivation would be bubbled with CO_2_ (3% in air) and supplied with elevated illuminations (150 μmol photons/m^2^/s). 0 μM (T0) and 500 μM (T500) theophylline were added to the culture broth.

With relatively weak illumination (50 μmol photons/m^2^/s) and limited carbon supply for the cells cultivated in flasks, the glycogen synthesis and storage in PCC 7942 cells were maintained at a relatively low level; thus, the effects of enhanced *glgP* expression and glycogen degradation activities might not be significant enough. We supposed that under conditions facilitating rapid glycogen accumulation, the effects of enhanced glycogen degradation activities might be more obvious; thus, we further evaluated the performance of the XC8 cells in column photobioreactors with bubbled 3% carbon-air gas. The illumination strengths were also elevated to 150 μmol photons/m^2^/s. As shown in Figure 3A, with enhanced illumination and carbon supply, both the wildtype and the XC8 strains grow significantly faster, with or without theophylline inductions, while the theophylline induction significantly reduced the final cell densities of XC8. During the cultivation process, intracellular glycogen contents of the wildtype were gradually increased, reaching from about 40 mg/gDCW on Day 3 to about 120 mg/gDCW on Day 6 and then were maintained on a stable level in the stationary phase; the addition of theophylline caused no influence on glycogen contents as well as cell growths (Figure 3B). As for XC8, when no theophylline was added, the glycogen contents would be maintained on a much higher level than those of the wildtype control, although the difference was declining with the prolonged cultivation process (about 5-fold on Day 3 and 1.75-fold on Day 6). When 500 μM theophylline was added, the glycogen contents would be reduced by about 70% to 60 mg/gDCW after 6 days of induction and cultivation, which is about 50% of the wildtype level on Day 6. The growth of the XC8 was reduced by the theophylline-induced *glgP* overexpression (Figure 3A). The final cell density was about 30% lower than that of the controls, indicating that the elevated glycogen turn-over rates might cause a non-significant cycle between glucose-1-phosphate and glycogen, leading to waste or inefficient utilization of the photosynthesis-derived energy and material flow.

### Enhanced *glgP* Expression Failed to Improve Intracellular Sucrose Accumulation of PCC 7942 Facing Salt Stress

**FIGURE 4:**
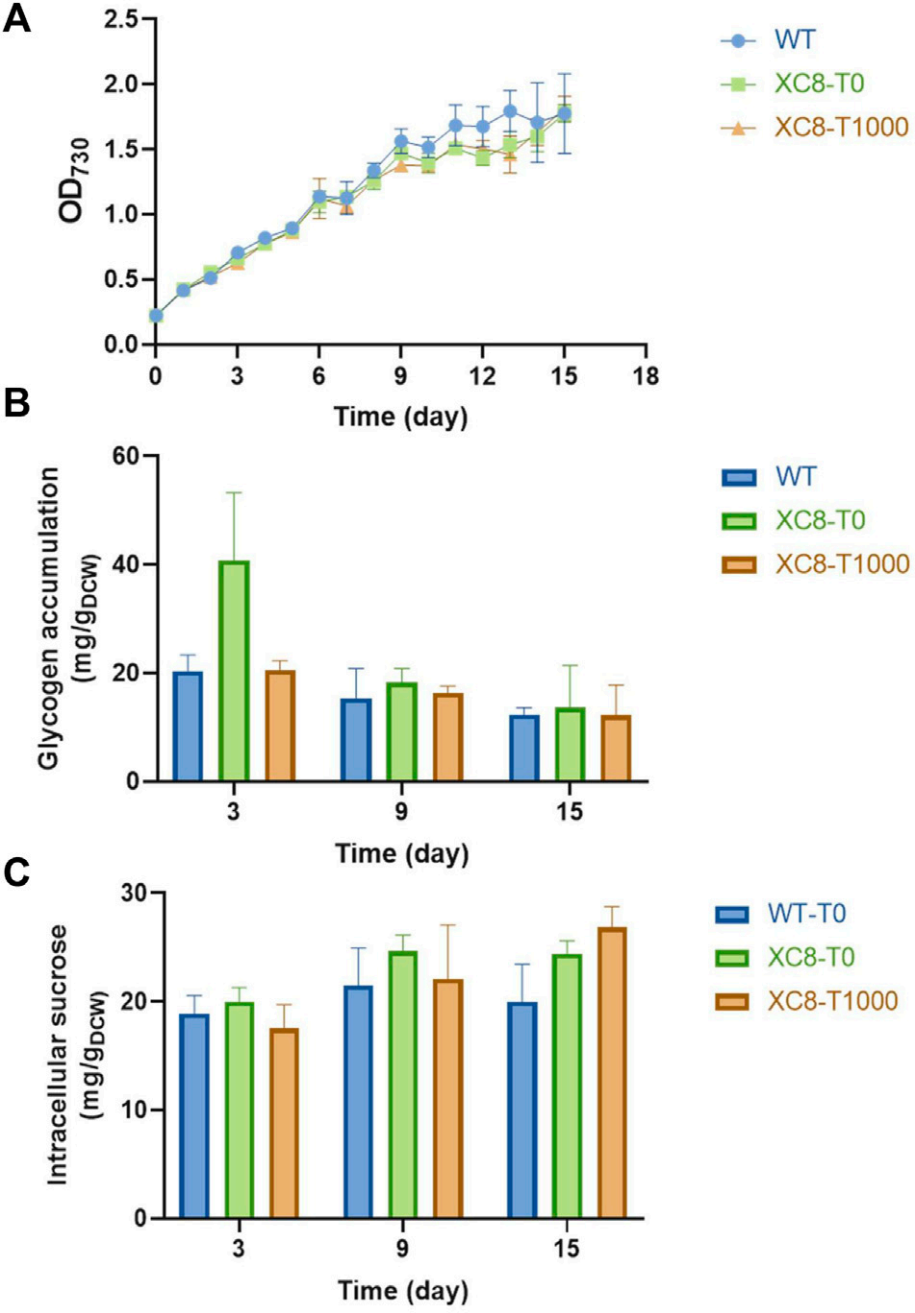
Effects of theophylline-regulated *glgP* expression on salt stress tolerance and intracellular sucrose storage in PCC 7942. **(A)** Growths of PCC 7942 wildtype and XC8 mutant strains facing 150 mM salt stress imposed on Day 3 with different doses of theophylline. **(B)** Glycogen storage of PCC 7942 wildtype and XC8 mutant strains facing 150 mM salt stress imposed on Day 3 with different doses of theophylline. **(C)** Intracellular sucrose concentrations of PCC 7942 wildtype and XC8 mutant strains facing 150 mM salt stress imposed on Day 3 with different doses of theophylline.

As the most important carbon sink mechanism in cyanobacteria, glycogen metabolism regulates and buffers the intracellular carbon flow distribution derived from photosynthesis. When treated with salt stress, carbon distribution in cyanobacteria cells would be rewired and the osmolyte synthesis would take up a significant portion of intracellular carbon, indicating potential interactions and competition with the glycogen metabolism network. Sucrose is the primary osmolyte synthesized in PCC 7942 to resist extracellular hypersaline conditions, and previously it has been reported that the blocking of the glycogen synthesis pathway would reduce the capacity to accumulate sucrose in PCC 7942 under hypersaline conditions and inhibit the salt tolerances (Miao et al., 2003; Suzuki et al., 2010). To explore the effects of enhanced *glgP* expression on sucrose accumulation of PCC 7942 facing salt stress, we imposed salt stress on PCC 7942 wildtype and XC8 strains supplemented with concentrations of theophylline. As shown in Figure 4A, when cultivating in hypersaline conditions, growths of XC8 were not influenced by the addition of theophylline to regulate GlgP abundances and glycogen storage. Although *glgP* expression in XC8 was significantly inhibited when no theophylline was added and the glycogen would be accumulated to a 2-fold higher level than that of the wildtype control, glycogen storage would still be dramatically reduced with the accumulation of intracellular sucrose under hypersaline conditions (Figure 4B). During the cultivation process with salt stress, glycogen contents in XC8 were maintained at the same level as that of wildtype control, with or without theophylline induction. However, the theophylline induction (1,000 μM) of *glgP* expression failed to elevate sucrose accumulation in XC8 cells. After 12 days of salt stressed cultivation, although intracellular sucrose concentration of XC8 cells was slightly higher than that of the wildtype control, no significant difference could be detected between the samples with or without theophylline induction (Figure 4C). Under hypersaline conditions, sucrose would be synthesized and accumulated in *Synechococcus* cells to resist the hyperosmotic stress and would be maintained at a favorable concentration rather than unlimited accumulation, which might be a possible explanation for the phenomenon that the reduction of glycogen contents failed to bring in elevated sucrose yield.

### Theophylline-Induced *glgP* Overexpression Failed to Enhance Salt Stress–Induced Secretory Sucrose Synthesis of PCC 7942 Cell Factories

Previously, it has been confirmed that the introduction of the *E. coli–*sourced cscB sucrose permease could facilitate the secretory synthesis of sucrose, which removes the potential intracellular over-accumulation effects and significantly improved sucrose production titers in cyanobacteria (Ducat et al., 2012; Song et al., 2016). Inspired by this, we explored whether the GlgP overexpression strategy could be used for enhancing secretory sucrose production in engineered cell factories.

**FIGURE 5:**
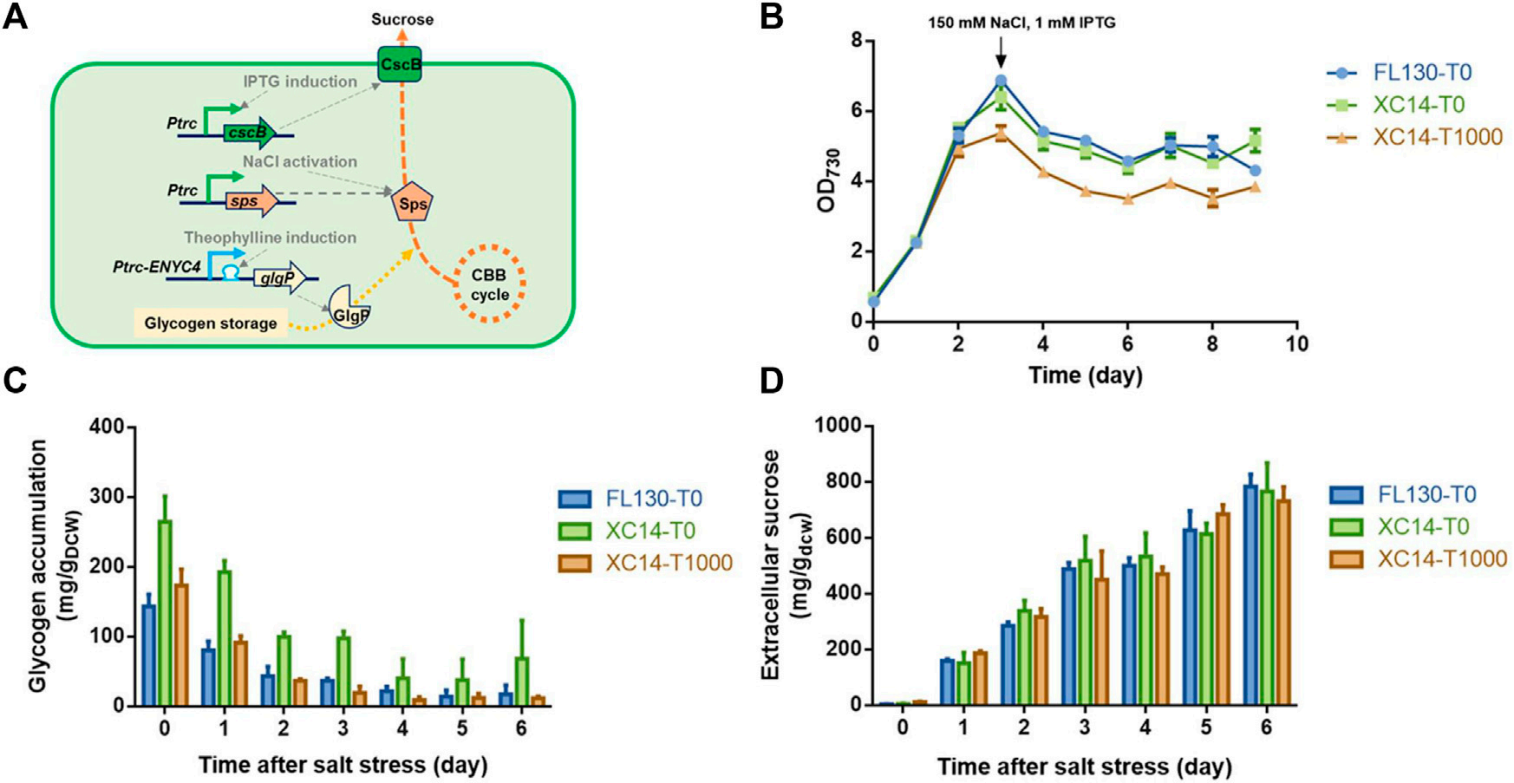
Effects of theophylline-induced *glgP* overexpression on salt-induced sucrose synthesis and secretion in PCC 7942 cell factories. **(A)** Design and working mode of the theophylline-regulated *glgP* expression system in a previously constructed sucrose-synthesizing strain FL130. The expression of the *cscB* gene would be induced by the addition of IPTG, facilitating sucrose secretion out of the cells. An additional copy of the native sucrose-6-phosphate synthase gene would be expressed by a constitutive strong promoter *Ptrc*, and when salt stress was imposed, the intracellular Sps would be activated by increased concentrations of NaCl, leading to enhanced sucrose synthesis with the carbon source from CBB cycle and glycogen storage. The promoter sequence of the native *glgP* gene would be replaced with the *Ptrc-ENYC4* system, and the addition of theophylline would lead to overexpression of *glgP* and enhanced glycogen degradation. The strain derived from FL130 was termed XC14. **(B)** Cell growths, **(C)** glycogen storage, and **(D)** extracellular sucrose production of FL130 and XC14 (with or without theophylline induction) strains before and after salt stress on Day 3. T0 and T1000 represented theophylline concentrations of 0 and 1,000 μM, respectively.

With a previously developed engineered strain FL130 carrying a heterologous *cscB* gene and an overexpressed native *sps* gene (encoding the sucrose-6-phosphate synthase, the rate-limiting enzyme catalyzing sucrose synthesis in PCC 7942), we utilized the *Ptrc-ENYC4* system to replace the native *glgP* promoter sequence and obtained the recombinant strain XC14 (Figure 5A). As shown in Figures 5B,C, when 1,000 μM theophylline was added to induce the overexpression of *glgP*, growth of XC14 would be inhibited, and the glycogen contents were reduced to a similar level to those of the FL130 control. After 150 mM NaCl was added to activate Sps and sucrose synthesis, glycogen storage in all the sets would be continuously decreased, indicating that a significantly rewired intracellular carbon distribution leads to enhanced sucrose production. After 3 days of cultivation under salt stress, glycogen contents in the XC14 strain without theophylline induction were still about 2-folds higher than those of the FL130 control and the XC14 cells with induction of 1,000 μM theophylline (Figure 5C). However, the difference in glycogen storage and *glgP* expressions caused minor effects on specific productivities of secreted sucrose (Figure 5D) on the per cell levels, and the final sucrose titer of the theophylline-induced XC14 strain was lower than that of the control due to the reduced cell growth. Comparing the wildtype and XC8 strains cultivated in flasks and column photobioreactors, the theophylline-induced *glgP* overexpression failed to improve the actual performances of the sucrose synthesis cell factories stressed by 150 mM NaCl. A possible explanation would be the low contribution ratio of glycogen storage in the salt-induced sucrose synthesis process. Due to the introduction of sucrose permease, the limitation of intracellular sucrose accumulation was removed, and a much larger portion of the carbon source would be rewired into sucrose synthesis, which would be then secreted to the extracellular environments. The carbon source from mobilized glycogen storage would take a much lower ratio for sucrose synthesis than that in the non-secretory mode. Thus, enhanced glycogen degradation could only bring in negligible contribution to carbon flow distribution, while the burden on protein overexpression and theophylline further caused the weakened performance of cell growth and sucrose titers, which is in accordance with the previously reported phenomenon by Ducat et al. (2012).

### GlgP Overexpression Improved Sucrose Synthesis in PCC 7942–Derived Cell Factories Under Non-Hypersaline Conditions

**FIGURE 6:**
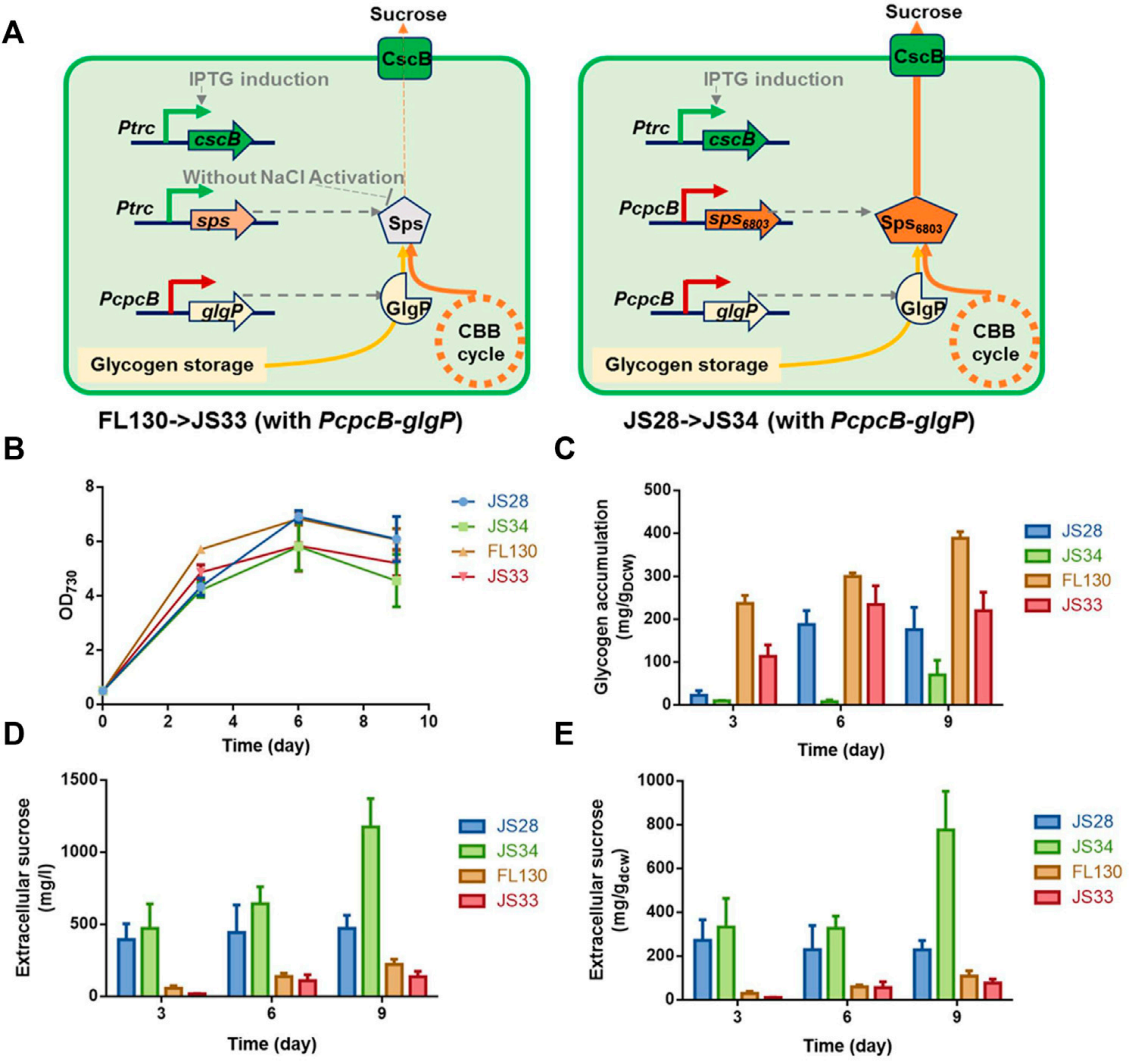
Effects of *glgP* overexpression on sucrose synthesis of PCC 7942 cell factories under non-hypersaline conditions. **(A)** To facilitate sucrose synthesis under non-hypersaline conditions, the PCC 6803 sourced *sps* gene *sps*_*6803*_ was put under the control of the *PcpcB* promoter to replace the *Ptrc* expressed native *sps* gene in FL130, and the obtained strain was termed as JS28. To enhance *glgP* expression, a *PcpcB* promoter–driven *glgP* expression cassette was introduced into FL130 and JS28, generating JS33 and JS34, respectively. Without salt stress induction, the Sps in FL130 and JS33 would be inactivated, and the sucrose synthesis would be inhibited. In JS28 and JS34, the NaCl-activation–independent Sps_6803_ could normally catalyze the sucrose synthesis process. Cultivated in column photobioreactors in standard conditions without salt stress, **(B)** cell growth, **(C)** glycogen contents, and **(D,E)** sucrose production of FL130, JS33, JS28, and JS34 cells were calculated in 9 days.

Based on the effects of theophylline-induced *glgP* expression on glycogen storage and sucrose synthesis in XC14 facing salt stress, we supposed that this strategy might more possibly be valuable in non-hypersaline conditions to link the glycogen degradation metabolism and synthesis of desired metabolites. As for PCC 7942, sucrose synthesis would be activated through salt ion–induced activation of sucrose phosphate synthase and inhibition of invertase (Liang et al., 2020), while it has been confirmed that Sps from PCC 6803 (hereafter termed as Sps_6803_ for short) was not a salt-activated enzyme, and introduction of the Sps_6803_ in the another *Synechococcus* strain UTEX 2973 successfully resulted in a secretory synthesis of sucrose without the need for salt stress induction (Lin et al., 2020). We took the same strategy to construct a PCC 7942–derived cell factory producing sucrose under non-hypersaline conditions by overexpressing Sps_6803_ in the FL92 strain which was previously developed and carried the same *cscB*-expression cassette as FL130 (Figure 6A), and the strain was termed as JS28 (*Ptrc-cscB*; *PcpcB-sps*_*6803*_). As shown in Figure 6B, growth patterns of the FL130, JS28, and the two respective glgP-overexpressing strains (JS33 and JS34) were quite similar under non-hypersaline conditions, while the glgP-overexpression caused a slight decrease of cell densities. As shown in Figure 6D, JS28 could synthesize about 500 mg/L sucrose, about 2.5 folds higher than that of the FL130 (*Ptrc-cscB*; *Ptrc-sps*) under the same non-hypersaline conditions. Glycogen storage in JS28 was also 50% lower than that of FL130, indicating that the secretory sucrose synthesis deprived a large portion of carbon flow from glycogen storage under non-hypersaline conditions (Figure 6C).

To explore the effects of enhanced glycogen degradation, we expressed *glgP* with a strong constitutive promoter *PcpcB* in both JS28 and FL130 (Figure 6A) and obtained the strains JS34 (*Ptrc-cscB*; *PcpcB-sps*_*6803*_; *PcpcB-glgP*) and JS33 (*Ptrc-cscB*; *Ptrc-sps*; *PcpcB-glgP*) respectively. As expected, glycogen storage in both of the two strains was reduced. In JS34, sucrose synthesis was correspondingly increased by about 2.4-folds compared with that of JS28 (with no *glgP* overexpression), reaching about 1,200 mg/L (accounting for up to about 40% of the fixed carbon, Figure 6E), while in JS33 (FL130 with *PcpcB* expressed *glgP*), the sucrose productivities and sucrose titers were not significantly increased compared with those of FL130, indicating that as for FL130 cells under non-hypersaline conditions, carbon flux was not the rate-limiting factor restricting sucrose synthesis.

Previously Ducat et al. have reported that combined overexpression of *glgP* and *cscB* could not improve sucrose productivities while causing a 5%–10% decrease (Ducat et al., 2012). In this work, we also evaluated the performances of similar strains JS33 (*Ptrc-cscB*; *Ptrc-sps*; *PcpcB-glgP*) cultivated under hypersaline conditions and got similar results when compared with the FL130 control (*Ptrc-cscB*; *Ptrc-sps*). As shown in Supplementary Figure S2, when cultivated under hypersaline conditions, expression of *glgP* reduced the sucrose titer from 2 g/L to 1.5 g/L after 9 days of cultivation. As mentioned above, the sharply reduced glycogen storage of salt-stress–induced sucrose-synthesizing cells provides a reasonable explanation for that phenomenon. As for FL130 strain facing continuous salt stress, a large portion of the carbon source (including the glycogen storage) has been motivated for sucrose synthesis; thus, the enhanced *glgP* expression and glycogen degradation activities could only bring in minor contributions but just consume more energy and resources on meaningless protein (GlgP) synthesis, which in turn causes impairment on the final sucrose yields.

The salt stress–responsive sucrose synthesis serves as a special case for cyanobacteria-based photosynthetic production, and the naturally evolved condition–induced metabolism shift spontaneously plays a role in regulating glycogen metabolism, and thus the artificially implemented glycogen motivation strategy could not bring in necessary effects. However, as for a majority of cyanobacteria metabolic engineering cases, the carbon sink in glycogen storage (Davies et al., 2014; van der Woude et al., 2014; David et al., 2018) and how to rewire the natural carbon sink into artificially assembled heterologous pathway was still an important issue to be solved. With the salt stress–independent sucrose synthesizing cell factories as a model, we have confirmed the feasibility of regulating the glycogen degradation pathway to engineer the glycogen metabolism and production capacities of the desired product.

## Materials and Methods

### Chemicals and Reagents

Unless noted otherwise, all reagents were purchased from Sigma-Aldrich (United States). Taq DNA polymerase and all restriction enzymes were purchased from Fermentas (Canada) or Takara (Japan). The kits used for molecular cloning were obtained from Omega (United States) or Takara (Japan). Oligonucleotides were synthesized, and DNA-sequencing was performed by Genewiz (Suzhou, China).

### Cyanobacterial Strains

All the cyanobacterial strains utilized in this work are listed in Table 1. To achieve theophylline dose–regulated *glgP* expression in PCC 7942 and FL130, a 155-bp DNA fragment, containing the *Ptrc* promoter and the theophylline-dependent riboswitch ENYC4, was synthesized according to the reported sequence (Nakahira et al., 2013). A *KmR* (kanamycin resistance gene) fragment, a 1.3-kb upstream fragment of the *glgP* gene (*Synpcc7942_0244*, according to the sequence from KEGG), the 155-bp *Ptrc-ENYC4* fragment, and a 1.0-kb *glgP* ORF (open reading frame) fragment were ligated in sequence (5′->3′) through infusion PCR, and the obtained fragment would be cloned into pMD18T (TaKaRa, Dalian, China) to get the pDY150 plasmid. The plasmid pDY150 would be transformed into PCC 7942 wildtype strain for construction of XC8 and into FL130 for the construction of XC14.

**TABLE 1:** Strain constructed and utilized in this work.

Strains^a^	Genotypes or characteristics	Sources
XC8	The cassette of “*KmR* ^b^ *-Ptrc-ENYC4*” was inserted between the *glgP* CDS and the native promoter sequence on chromosome of the PCC 7942 wildtype strain.	This work
FL92	*NS3::Ptrc-cscB-CmR* ^b^	Qiao et al. (2018)
FL130	*NS1::Ptrc-sps-SpR* ^b^ *; NS3::Ptrc-cscB-CmR*	Qiao et al. (2018)
XC14	The cassette of “*KmR-Ptrc-ENYC4*” was inserted between the *glgP* CDS and the native promoter sequence on the chromosome of FL130 strain.	This work
JS28	*NS2::PcpcB-sps*_*6803*_ *-SpR*; *NS3::Ptrc-cscB-CmR*	This work
JS33	*NS1::Ptrc-sps-SpR*; *NS2::PcpcB-glgP-GmR* ^b^; *NS3::Ptrc-cscB-CmR*	This work
JS34	*NS1::PcpcB-glgP-GmR*; *NS2::PcpcB-sps*_*6803*_ *-SpR*; *NS3::Ptrc-cscB-CmR*	This work

a All the listed strains are derived from *Synechococcus elongatus* PCC 7942, which is a gift from Prof. Xudong Xu’s Lab in the Institute of Hydrobiology, Chinese Academy of Sciences.

b *KmR*, kanamycin resistance gene; *CmR*, chloramphenicol resistance gene; *SpR*, spectinomycin resistance gene; *GmR,* gentamicin resistance gene.

To achieve sucrose synthesis under non-hypersaline conditions in PCC 7942, the PCC 6803-sourced *sps* gene (*sll0045*, according to the sequence from KEGG, termed as sps_6803_) would be cloned and introduced into FL92. The backbone of a previously constructed plasmid pQL225 (Qiao et al., 2018), containing upstream and downstream homologous fragments of neutral site 2 (NS2) on the chromosome of PCC 7942 and the *PcpcB* promoter sequence, would be amplified and fused with the *sps*_*6803*_ fragment and the spectinomycin-resistance gene (*SpR*) fragment using the Seamless Assembly Cloning Kit (CloneSmarter, C5891) to generate the plasmid pJS13. The pJS13 plasmid would be transformed into FL92 for construction of JS28.

To enhance *glgP* expression in FL130 and JS28, the native *glgP* gene from PCC 7942 would be amplified. The backbone of a previously constructed plasmid pQL225 (Qiao et al., 2018) containing upstream and downstream homologous fragments of neutral site 2 (NS2) on the chromosome of PCC 7942, the *PcpcB* promoter sequence, and the gentamicin resistance gene (*GmR*) fragment would be amplified and fused with the *glgP* fragment to generate the plasmid pJS23. The backbone of another previously constructed plasmid pFL20n (Duan et al., 2016) containing the upstream and downstream homologous fragments of neutral site 1 (NS1) on the chromosome of PCC 7942 would be amplified and ligated with the *PcpcB-glgP-GmR* fragment from the pJS23, and the generated plasmid was termed as pJS22. Plasmids pJS22 and pJS23 were transformed to the strains JS28 and FL130, respectively, for the construction of JS34 and JS33.

*Escherichia coli* DH5α was used as the host for the construction of plasmids. The respective plasmids would be transformed into *Synechococcus* cells, and the antibiotic-resistant transformants were usually obtained after 7–10 days of cultivation on selective BG11 agar plates. The genotypes of the transformants were verified by PCR and DNA-sequencing.

### Cyanobacteria Cultivations

The wildtype and engineered *Synechococcus* strains were cultivated with BG11 medium (Rippka et al., 1979) in 250-ml flasks or 350-ml column photobioreactors (580 mm by 30 mm). The cultivation with flasks would be performed in a horizontal rotary shaker with 130 rpm under moderate intensity (30–50 μmol photons/m^2^/s) provided by white-light illumination lamps. When the column photobioreactors were utilized, 3% CO_2_ (V/V in the air) would be bubbled to provide a carbon source, and the illuminations would be elevated to about 150 μmol photons/m^2^/s. As for the recombinant strains, concentrations of antibiotics (20 μg/ml spectinomycin, 20 μg/ml kanamycin, 10 μg/ml chloramphenicol, and 2 μg/ml gentamicin) would be added to BG11 medium when required. When multiple antibiotics are used at the same time, the concentration of each antibiotic would be reduced. During the cultivation process, cell growths were calculated by measuring the optical density at a wavelength of 730 nm (OD_730_) and converted to dry cell weight (DCW) with a pre-established calibration between the OD_730_ and DCW of PCC 7942 cultures (1.0 OD_730_ unit equals approximately 0.34 gDCW/L). To regulate glycogen expression or to induce sucrose synthesis, theophylline, IPTG, and NaCl would be supplemented as necessary. Concentrations of theophylline were selected based on previous experience with the *Ptrc4-ENYC4* element (Qiao et al., 2018; Chi et al., 2019). At least three biological replicates were performed for each experiment to ensure repeatabilities.

### Western Blot for GlgP Abundances

*Synechococcus* cells were harvested by centrifugation at 4°C. The resuspended cells (in 50 mM Tris-HCl buffer, pH 8.0) were disrupted with 100-mm glass beads (Sigma) in an ice bath. After removing the cell debris and glass beads by 4°C centrifugation, the supernatants were collected. Protein concentrations of the cell-free extracts were also measured with the Bradford method. The protein samples were analyzed on 12% SDS-PAGE with a standard procedure and blotted onto PVDF membranes, sealed with 5% nonfat milk-TBST buffer (TBS added with 0.05% Tween-20) at 4°C overnight. First, the membrane was incubated with polyclonal antibodies to GlgC (1:1000) (Hangzhou HuaAn Biotechnology Co., Ltd., Hangzhou, China) for 3 h and washed three times with TBST (15 min each time). Second, the membrane was incubated with an alkaline phosphatase–linked secondary antibody (goat anti-rabbit, Invitrogen, Shanghai, China) for 1 h and washed three times with TBST (15 min each time). Finally, the membrane was developed with BCIP/NBT (Sigma).

### Determination of Sucrose Contents

To evaluate the sucrose production of the strains constructed in this work, the culture broth would be sampled and centrifuged at 10,000 g. To determine the extracellular sucrose amounts, the supernatants would be analyzed with the Sucrose/D-glucose Assay Kit (Megazyme, K-SUCGL). To calculate the intracellular sucrose concentrations, the pellet would be resuspended with 80% (V / V) ethanol and heated at 65°C for 4 h. After being centrifuged at 15,000 g for 5 min, the supernatant would be carefully collected and completely dried by blowing of N_2_ stream at 55°C. Finally, the dried samples would be resuspended with ddH_2_O and assayed with the Sucrose/D-glucose Assay Kit.

### Glycogen Phosphorylase Activities Determination

Glycogen phosphorylase activities of the *Synechococcus* strains were calculated following the previously introduced protocols with modifications (Fu and Xu, 2006). The cells were resuspended and disrupted in buffer A (containing 18 mM KH_2_PO_4_, 27 mM Na_2_HPO_4_, 15 mM MgCl_2_, 100 μM EDTA, pH 6.8). A 200-μl reaction system for assay contained 30 μl crude enzyme, 18 mM KH_2_PO_4_, 27 mM Na_2_HPO_4_, 15 mM MgCl_2_, 100 μM EDTA, 340 μM Na_2_NADP (Amresco), 4 μM glucose-1,6-biphosphate (Santa Cruz), six units ml-1 glucose-6-phosphate dehydrogenase (Sigma), 0.8 units ml-1 phosphoglucomutase (Sigma), and 2 mg ml-1 glycogen (Sigma). The same system lacking glycogen was used as a control. GlgP activities were measured by calculating the generation speeds of NADPH with a change in absorbance at 340 nm. The calculations of Abs340 were performed with 96-well plates by a microplate reader. The reactions were initiated by adding the crude enzyme.

### Glycogen Determination

Intracellular glycogen contents would be calculated as previously described (Grundel et al., 2012), with minor modifications. *Synechococcus* cells would be collected by centrifugation at 10,000 g for 15 min. The pellet would be washed three times with ddH_2_O, resuspended with 30% (W/V) KOH, heated at 95°C for 2 h, and finally, ice-cooled ethanol would be added to a final concentration of 70%–75% (V/V). The mixture would be cooled at -20°C overnight and then centrifuged at 15,000 g for 15 min to collect the glycogen precipitation. The glycogen pellet would be washed twice with 70% ethanol (V/V) and 98% ethanol (V/V) successively and then dried by vacuum centrifugation. The glycogen finally obtained was suspended in 100 mM sodium acetate and digested by amyloglucosidase (Novozymes). The glucose contents generated in the glycogen solution were determined with the Sucrose/D-glucose Assay Kit.

## Data Availability

The raw data supporting the conclusion of this article will be made available by the authors, without undue reservation.
