# Supplementary material for: Manipulating the Expression of Glycogen Phosphorylase in Synechococcus elongatus PCC 7942 to Mobilize Glycogen Storage for Sucrose Synthesis
Source: Front Bioeng Biotechnol. 2022 Jul 1;10:925311. doi: 10.3389/fbioe.2022.925311 (PMC9284946; doi:10.3389/fbioe.2022.925311)
Supplement: Supplementary file 1 [file Image1.pdf]

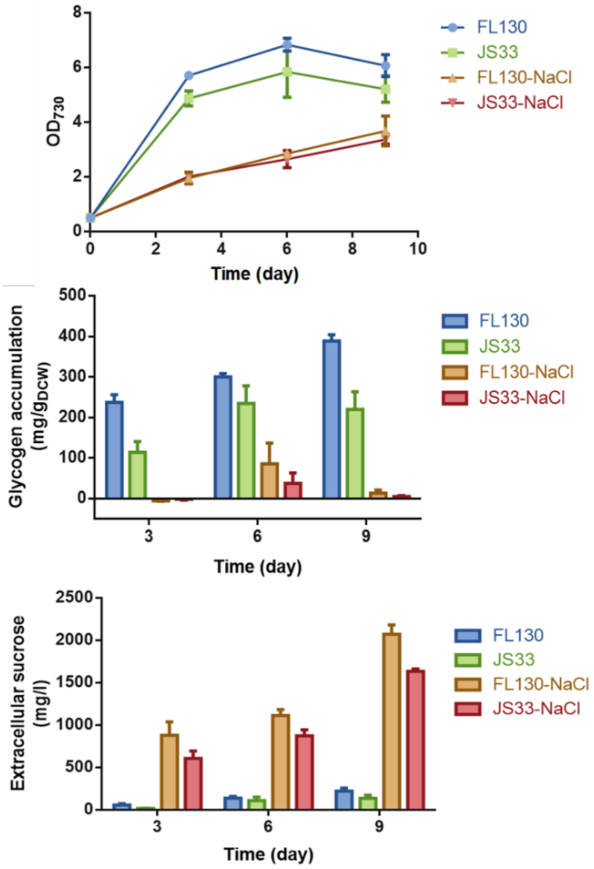

Figure S1. Effects of glgP overexpression on cell growth, glycogen accumulation, and sucrose synthesis in FL130 with or without salt stress.
